# Personalized Diabetes Therapy Part 1—Functional Phenotyping as a Conceptual Basis for Individualized Treatment

**DOI:** 10.3390/jpm16040226

**Published:** 2026-04-18

**Authors:** Andreas Pfützner, Julia Jantz

**Affiliations:** 1 Pfützner Science & Health Institute, D-55128 Mainz, Germany; julia.jantz@pfuetzner-mainz.com; 2 Institut for Digital Technology in Medicine & Dentistry, Institut Supérieur der Formation Continue, L-5359 Schuttrange, Luxembourg; 3 Department of Biotechnology & Bioinformatics, Technical University Bingen, D-55411 Bingen, Germany

**Keywords:** precision medicine, personalized diabetes care, phenotyping, intact proinsulin, adiponectin, hsCRP

## Abstract

The diagnosis of type 2 diabetes using classical clinical and laboratory biomarkers (HbA1c, glucose, lipids, BMI, and blood pressure) is a classification by symptoms and does not provide insight into the underlying pathophysiological disorders (insulin resistance, β-cell dysfunction, visceral adipose tissue hormonal secretion, and chronic systemic inflammation). A better understanding of these disorders may help in the selection of appropriate and potentially more successful personalized therapeutic interventions. Based on extensive clinical trial experience, a method for individual phenotyping and consecutive personalized diabetes therapy has been developed in our practice, which we have been using for more than 15 years and would like to share for discussion and debate. In this Part 1, the pathophysiological background and diagnostic approach to phenotyping is described. A consecutive Part 2 will present the translation of the phenotyping result into a personalized diabetes therapy, and another consecutive Part 3 will provide more comprehensive real-world patient observations when practicing this concept. This article is intended as a discussion/concept paper and does not present unpublished patient-level outcome data or formal effectiveness analyses. Prospective validation studies are needed to evaluate the clinical utility of this phenotype-based framework.

## 1. Introduction

Type 2 diabetes is a highly complex multifactorial disease consisting of several interrelated underlying disorders (β-cell dysfunction, insulin resistance, hormonal hyperactivity of the visceral adipose tissue, and chronic systemic inflammation). This diagnosis has been made for centuries exclusively on the basis of urine and blood glucose elevation, which ultimately only represents a symptom of the disease and only captures the clinical picture very superficially [1,2]. Today, type 2 diabetes has become a major global health problem. According to the International Diabetes Federation, 589 million adults aged 20–79 years were living with diabetes worldwide in 2024, and this number is projected to rise to 853 million by 2050 [3]. Most cases are type 2 diabetes and are closely linked to the increasing prevalence of overweight and obesity, particularly visceral adiposity, which promotes insulin resistance and chronic low-grade inflammation [4,5]. Beyond hyperglycemia, diabetes imposes a substantial burden through cardiovascular, renal, neurological, ophthalmological, and foot complications, which remain major causes of disability and premature mortality worldwide [3,6]. The current guideline-based therapy with an almost exclusive focus on the normalization of blood glucose and its surrogate parameter HbA1c [2] has led to the impression that type 2 diabetes mellitus is a chronic progressive disease. The majority of patients die from macrovascular or microvascular events, which appear to be practically unavoidable even if glycemic treatment goals are achieved [7,8,9].

The conceptual framework described here was developed from clinical and research experience at the Pfützner Science & Health Institute, Mainz, Germany. It is based on more than 30 years of clinical and study experience, and is presented here for scientific discussion rather than as a formal proof-of-effectiveness model. The present article should therefore be interpreted as a perspective/concept paper. It does not report new prospective outcome data or comparative effectiveness analyses. Instead, it seeks to place a practice-based functional phenotyping framework into the current debate on individualized diabetes care and precision medicine. On the basis of extensive study experience, a method for phenotyping and consecutive personalized diabetes therapy has been developed at our institute, which we would like to present here and put up for international discussion. Important and first of all: this discussion paper has no missionary background. We would like to present and describe an individualized approach to diabetes therapy, which is based on more than 30 years of clinical and study experience (>400 clinical trials). We have applied this framework in routine practice for many years and herein present it as a discussion model for scientific debate, not as a formal report of effectiveness outcomes.

This Part 1 describes the background and procedure for phenotyping with functional biomarkers. A consecutive Part 2 presents the translation of phenotyping results into an individualized diabetes therapy, and we will show real-world patient examples treated according to this concept in our practice in Part 3. The basis for our personalized treatment concept is our understanding of the disease pathophysiology, as described in the next section.

## 2. Physiological Background

**Figure 1:**
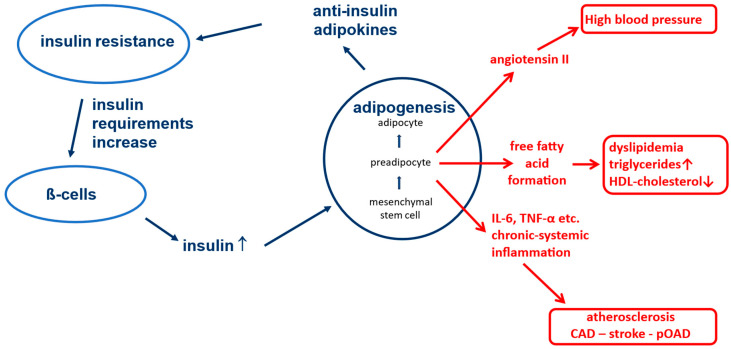
Physiological processes during weight gain and associated possible pathological consequences.

The biological processes of energy balance of the human organism, which form an important basis for the pathophysiology of type 2 diabetes, represented a survival advantage many thousands of years ago. It can be assumed that in the Stone Age, when our ancestors still lived in caves and hunted prehistoric animals, people did not always have regular and sufficient access to food. This gave individuals a survival advantage—and they prevailed genetically—if they were able to store as much energy as possible as lipid tissue when there was an abundance of food so that they could draw on it in times of starvation. Physiologically, the formation of adipose tissue in the body is the exclusive domain of insulin action [10]. Insulin is known to stimulate the differentiation of mesenchymal stem cells into preadipocytes (see Figure 1) [11,12,13].

These preadipocytes, in turn, are the source of numerous so-called “adipokines”, which, despite their highly diverse effects in the body, have one biochemical property in common: they all act against insulin at different receptors and/or cellular levels [14,15,16]. This results in a metabolic insulin resistance and increased insulin requirement for glycemic control. The production capacity of pancreatic β cells is very high, and consequently more insulin is produced in response to increased need, which supports further differentiation of mesenchymal stem cells into preadipocytes. Ultimately, these physiological relationships allow the body to tolerate more insulin and use it to generate lipid tissue for energy storage without experiencing negative effects on blood glucose levels. After all, an unconscious person in hypoglycemia cannot eat. In fact, at least in the western world, we are currently living in times of permanent food abundance. As a consequence of these evolutionary conditions, the world has been experiencing a wave of obesity for several decades that has never been seen before in the history of humankind [17].

Adipokines, among other properties, may have negative effects on blood pressure. A prominent representative is angiotensin II, which is formed and released in an uncontrolled fashion by preadipocytes and drives up blood pressure [18]. Also, some adipokines induce the formation of free fatty acids and triglycerides. Hypertriglyceridemia and low HDL levels therefore often occur in this situation [19]. The already increased cardiovascular risk due to high blood pressure and dyslipidemia (see Figure 1) place associated fatal diseases (heart attack, stroke, etc.) among the main causes of death in the western world, even independently of diabetes [20]. A reduction in vaso-protective insulin effects (e.g., a reduction in anti-oxidative nitric oxide secretion) [21]) is an additional contributing factor to this negative outcome.

Another factor that increases macrovascular risk is the development of chronic activation of the immune system (chronic systemic inflammation) on the basis of stem cell differentiation in adipose tissue [5,22,23]. Whenever differentiation processes take place in the body, there is also a very small amount of incomplete differentiation of stem cells with an occasional risk of formation of cancer cells. To prevent these from causing damage, the immune system is alerted, and activated macrophages migrate into the fatty tissue, which can recognize and destroy mutated cells [24,25]. However, as the immune system cannot be activated only locally in a single tissue, all monocytes/macrophages in the body are activated in this situation, including immune cells circulating in the vasculature [26,27]. These cells may have occasionally taken up oxidized LDL cholesterol, e.g., in cases of hypercholesterolemia [28,29]. The penetration of these activated lipid-laden monocytes/macrophages into the vessel wall induced by other trigger mechanisms (e.g., hypertension, hyperglycemia, etc.) is the immunological basis for the development of atherosclerosis [30].

The correlations described above already give an idea of why people who are overweight, especially during weight gain, are prone to hypertension, lipid disorders, insulin resistance and atherosclerosis [31]. The situation becomes even more serious when an inherited type 2 diabetes mellitus is developing, which turns this physiology into the pathophysiology of a complex metabolic and vascular disease.

## 3. Pathophysiological Aspects of Type 2 Diabetes Mellitus

According to all available current genetic studies, type 2 diabetes mellitus is mainly due to hereditary disorders of β-cell dysfunction [32,33]. While the general public community often assumes that an unhealthy lifestyle leads to diabetes, it can be taken from the entire literature that only individuals who carry certain genes associated with β-cell dysfunction ultimately develop type 2 diabetes [34]. Otherwise, subjects become obese and probably develop orthopedic problems as well as cardiovascular symptoms at some point. In our experience, however, a healthy lifestyle can significantly delay the manifestation of diabetes.

**Figure 2:**
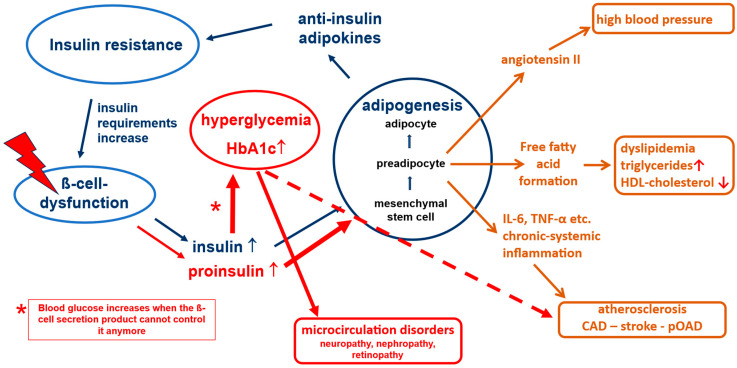
Pathophysiological processes in type 2 diabetes.

In type 2 diabetes, several secretion disorders of β cells are in the foreground. According to current data, the physiological pulsatility of insulin secretion and the first insulin response to the meal fail as first indications for diabetes (timing secretion disorder) [35,36,37]. Instead of releasing an insulin peak six times per hour, insulin secretion takes place in a “steady flow” (“stage I of β-cell dysfunction” [38,39]). As a result, the protective effect of insulin in microcirculation fails [21]. This could be one reason why people with diabetes who have very good glycemic control can develop microcirculatory disorders after sufficiently long disease duration, especially if other risk factors are also present [40]. Due to the insulin resistance-mediated increased insulin requirement, “hyperinsulinemia” (quantitative secretion disorder, stage II of β-cell dysfunction) is known to occur [41], which subsequently leads to exhaustion of the cleavage capacities of β cells and consecutively to a release of proinsulin in addition to insulin (qualitative secretion disorder; stage III of β-cell dysfunction) (Figure 2) [41].

Proinsulin, the precursor of insulin in the cellular insulin production process, is a non-physiological hormone that can also lower blood sugar, but only with 10–20% of the effectiveness of insulin [41]. At the same time, it has the same lipogenetic action as insulin [12]. On a molecular level, 5 to 10 times more proinsulin than insulin is needed to control blood glucose, which can in turn further increase the negative effects of adipokines on the body. In this situation, the blood glucose-lowering effect of proinsulin can help to maintain normoglycemia, while the other pathological processes continue unabated. In this phase, a competitive race takes place in the body: is there still enough time for the (blood glucose-based) diagnosis of diabetes mellitus, or does a fatal macrovascular event occur beforehand? The correlations described also explain why manifest atherosclerotic vascular changes are already diagnosed in many affected people when type 2 diabetes mellitus is first diagnosed [2,42].

Hyperglycemia in turn increases the insulin demand and further boosts insulin production, which accelerates the vicious circle of this pathophysiology. This underlines the need for good blood glucose control. If blood glucose is not consistently normalized, glucose toxicity leads to a significant acceleration in the development of microvascular and macrovascular complications. Our approach to achieve a stable and non-progressive long-term glycemic control is to treat with lifestyle and pharmaceutical drugs and/or drug combinations guided by a panel of functional biomarkers determining the individual degree of severity of the underlying disease deteriorations. It is of note that usually one of the deteriorations can be the predominant “driver” of the disease pathology, which can also lead to different clinical appearances (phenotypes).

## 4. Why Phenotyping?

**Figure 3:**
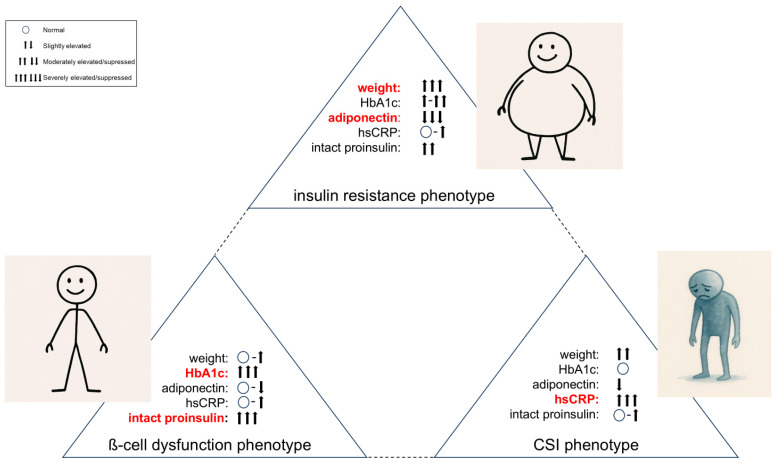
Functional (extreme) phenotypes in type 2 diabetes (leading biomarker indicators are highlighted in red).

The basic disorders described above (β-cell dysfunction, insulin resistance, visceral adipose tissue activity, chronic systemic inflammation—hereafter “CSI”) can be present in different stages and with different degrees of severity, which leads to different clinical phenotypes (Figure 3). The phenotype, which is primarily driven by β-cell dysfunction (BCD-Phenotype), may, in our clinical experience, be less satisfactorily addressed by standard stepwise glucocentric treatment approaches. The people affected are often rather slim or only slightly overweight and usually have poor blood glucose and HbA1c values until insulin is finally used after guideline-compliant therapy escalation.

The insulin resistance-driven phenotype (IR-Phenotype) presents significant obesity, difficult-to-treat hypertension and often has already manifest and systemic atherosclerosis.

One extreme phenotype is certainly the normoglycemic person with “cardiodiabetes”, who is often slightly overweight and presents clinically with arterial hypertension, dyslipidemia and hyperuricemia (CSI-driven phenotype). This phenotype is currently not recognized as diabetes-related disease, as it does not fulfill the glucose criteria for diabetes diagnosis. It is therefore only treated symptomatically.

**Figure 4:**
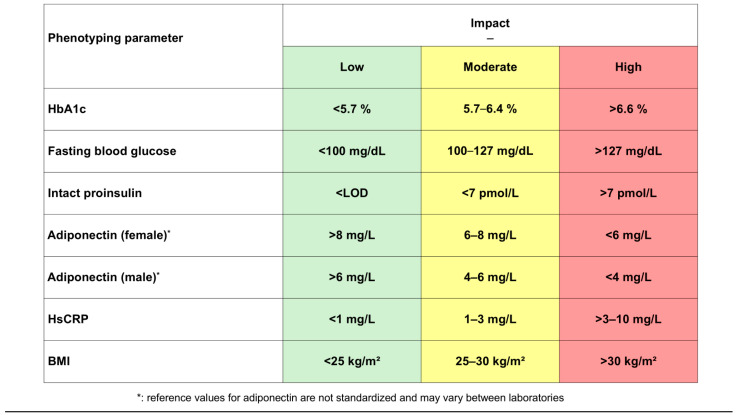
Phenotyping biomarkers (LOD: limit of detection).

In our experience, the phenotype of each person with diabetes lies individually between these three extremes and it is difficult to consider that a glucocentric and solely HbA1c-fixed escalating standard approach (diet and lifestyle → metformin → metformin + another antidiabetic drug → metformin + two other antidiabetic drugs → insulin +/− other antidiabetic drugs, [2]) should be able to treat these diverse clinical pictures so efficiently that microcirculatory complications and macrovascular events can be prevented. In any case, according to current data, guideline-based HbA1c-controlled standard therapy does not really lead to a reduction in the main causes of death in people with diabetes (heart attack and stroke). Even when HbA1c treatment targets are achieved, it is known that diabetes usually progresses and often leads to final macrovascular endpoints [7,8,9]. The rationale of our personalized treatment approach is to address these underlying pathophysiological processes more directly than a solely glucocentric strategy does. Our approach is based on a panel of biochemical and clinical parameters as provided in Figure 4.

## 5. How to Phenotype Effectively?

The classification of patients with type 2 diabetes based on the classic clinical and laboratory markers (HbA1c, glucose, BMI in context with lipids and blood pressure) is a classification according to symptoms and provides virtually no insight into the underlying pathophysiological disorders (insulin resistance, β-cell dysfunction, adipogenesis and CSI). Our biomarker concept (without association to phenotypes) as shown in Figure 4 was published in 2008 and has been employed in our practice for more than 15 years now [43].

The assessment of β-cell dysfunction is of particular interest to us, as more and more drugs have been developed to protect β cells or maintain their functionality, such as GLP-1 analogs or DPPIV inhibitors.

In addition to conventional means of assessing β-cell function and insulin resistance (e.g., HOMA score or meal-related insulin/C-peptide secretion), we use the determination of intact proinsulin (iPI) in the fasting state or under stress to determine the extent of β-cell dysfunction and macrovascular risk. iPI, together with insulin or C-peptide, is an indicator of the overall remaining production capacity and production quality of β cells. There are numerous reports from randomized, prospective long-term studies with large cohort numbers proving that iPI is not only a valid risk indicator for an imminent diabetes manifestation or for macrovascular events review in [44], but must even be regarded as a cardiovascular risk factor. When proinsulin binds to insulin receptors at the vessel wall, this leads to atherogenic activation of MAP kinase in the endothelium with the release of atherogenic inflammatory factors (e.g., endothelin I) [12]. Treatment with high doses of intact proinsulin in the context of earlier pharmaceutical product development led, among other things, to massive and uncontrolled secretion of plasminogen activator inhibitor 1 (PAI-1) from visceral adipose tissue [45]. This cytokine is known to block the physiologically necessary thrombolysis [46,47]. After several unexplainable and two fatal macrovascular events occurred in patients with new-onset type 1 and type 2 diabetes during phase II of this drug’s development, the development of proinsulin as an antidiabetic agent was discontinued [41].

The determination of an elevated fasting iPI in a patient with normal glucose values is in any case indicative of β-cell dysfunction and clinically relevant insulin resistance [48], especially in the CSI-driven phenotype, which can be diagnosed, for example, by measuring a high iPI with normal glucose levels. In terms of differential diagnosis, elevated fasting iPI values otherwise only occur in the case of a (very rare) proinsulinoma [49,50] or the early manifestation phase of type 1 diabetes [51].

Adiponectin is another physiologically important adipokine, although it is produced in mature adipose tissue (i.e., not in preadipocytes) and in connective tissue. It is known to increase insulin sensitivity in the liver and periphery and to have vaso-protective and anti-atherosclerotic effects [43,52,53]. In visceral adipogenesis, adiponectin secretion is suppressed, which leads, for example, to a further increase in insulin resistance [54]. In our concept, the suppression of adiponectin is an indicator of the extent of anti-insulin visceral hormonal activity and the lead biomarker in the insulin resistance-driven phenotype.

A detailed analysis of the Framingham study cohort by Ridker et al. showed that CRP concentrations in the near-normal range (<10 mg/dL) allow independent stratification of cardiovascular risk into three risk groups when measured with a highly sensitive test method (hsCRP) [55,56,57]. This marker has gained worldwide acceptance as a biomarker of chronic systemic inflammation and is part of the risk assessment guidelines of many scientific societies, including the American Heart Association and the American Diabetes Association [2,58]. Values below 1 mg/L describe a low cardiovascular risk, 1–3 mg/L indicate a moderate cardiovascular risk, and 3–10 mg/L describe a population at high risk. Values above 10 mg/L may occur due to other non-specific infections and inflammation, and therefore cannot be used to assess the chronic systemic vascular inflammatory process [43,55,56,57,58]. In type 2 diabetes, elevated hsCRP levels are often associated with marked insulin resistance and substantial β-cell dysfunction [59].

**Figure 5:**
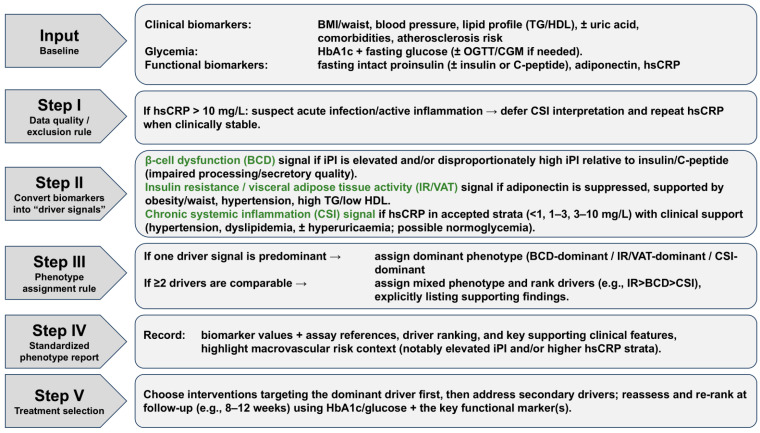
Workflow of the phenotyping process.

With this biomarker information and with consideration of the clinical appearance of the patient, this framework may help guide treatment decisions for underlying disorders rather than treating the symptom of elevated glucose of elevated HbA1c. A description of the stepwise workflow of the phenotyping process is provided in Figure 5.

It is of note that mature onset of diabetes in the young (MODY) and late onset of autoimmune diabetes in the adult (LADA) are important differential diagnoses because they are frequently misclassified as type 2 diabetes. It is therefore of importance to rule out MODY and LADA before applying phenotype-based therapy. We have occasionally encountered such cases in clinical routine. In suspected LADA, we use autoantibody testing (e.g., GAD65 ± IA-2/ZnT8, as available) and assess endogenous insulin reserve. OGTT-based insulin/C-peptide responses are typically reduced compared with type 2 diabetes [60]. In the glucose challenge test, we have observed decreasing 2 h intact proinsulin values in LADA patients, while stable or increased 2 h levels are typically seen in type 2 patients, but this observation requires further evaluation. We consider MODY genetic testing particularly in patients with features such as diabetes in multiple successive generations suggestive of autosomal dominant inheritance, diagnosis at a young age, absence of obesity/metabolic syndrome features, preserved C-peptide and negative islet autoantibodies, atypical response patterns to standard T2D escalation, or unexpectedly high sensitivity to sulfonylureas in appropriate contexts [61,62].

## 6. “Blood Glucose Cosmetics” vs. Phenotype-Driven Personalized Therapy

The pathophysiological relationships described above raise the question how an exclusive therapeutic focus on blood glucose and HbA1c can ultimately improve macrovascular prognosis. Therapeutic guidelines worldwide always recommend starting treatment for type 2 diabetes with diet and lifestyle measures followed by using metformin as the first line drug [2,63]. This is justified by all professional societies with the evidence base regarding the development of late complications in comparative studies. However, it ignores the fact that there are practically no direct comparative studies between metformin and modern antidiabetic drugs. Non-specific insulin secretagogues (sulfonylureas, glinides) reduce blood glucose efficiently, especially at the beginning of therapy, but at the same time may not adequately address, and in some settings may potentially aggravate, key pathophysiological mechanisms. There is—in our opinion—more than sufficient evidence in the literature that their consistent prescription and use by patients leads to a significant increase in cardiovascular risk [64,65,66]. To a certain extent, they may be associated with unfavorable downstream effects; in a direct comparison with sulfonylureas, metformin therefore performed significantly better [67].

As a result, metformin has achieved the currently undisputed status of “first-line” medication despite its well-known very high gastrointestinal side-effect profile. However, its mechanism of action (inhibition of hepatic gluconeogenesis from adipose tissue) does not directly address all the proposed underlying pathophysiological domains, and often even actively counteracts weight loss. In fact, it shows only a moderate effect on weight loss in current meta-analyses [68]. In one of the few direct randomized prospective mono-therapeutic head-to-head comparative studies against a pathophysiologically-oriented antidiabetic agent (rosiglitazone) in the ADOPT study [69], metformin monotherapy was superior to glimepiride (sulfonylurea) in terms of inhibition of diabetes progression—measured over the time until the need for an additional antidiabetic agent—but performed significantly worse than rosiglitazone. In a direct comparison with dulaglutide in the AWARD-3 study, metformin also showed poorer treatment results [70]. It can, therefore, not be expected that the progression of type 2 diabetes can generally be prevented with metformin as a first-line therapy.

This paper has several limitations. It is not a formal systematic review and does not provide new prospective outcome data, statistical validation, or comparative effectiveness analyses. The proposed phenotyping model is therefore hypothesis-generating and should be tested in appropriately designed observational and interventional studies. In addition, access to functional biomarker testing may vary substantially across healthcare systems, and in some settings reimbursement, local laboratory infrastructure, or turnaround times may restrict the routine availability of markers such as intact proinsulin (iPI), adiponectin, or high-sensitivity CRP beyond standard glucose/HbA1c and lipid panels. Limited access could reduce the precision of phenotyping and may delay therapy adjustments. Therefore, broad implementation requires adaptation to local resources and may need stepwise integration. Accordingly, the present article should be interpreted as a perspective/concept paper rather than as a formal review or original research report.

In summary, type 2 diabetes is a highly complex disease driven by several interlocking underlying disorders, which can be present in varying degrees of severity. This can lead to very individual clinical phenotypes, none of which are currently optimally treated by standardized glucocentric therapy. The method of phenotyping using functional biomarkers, which we have described in this discussion paper, and which has been used as a practice-based conceptual framework for more than 15 years, may support a more pathophysiology-oriented and individualized therapeutic approach for individual and personalized diabetes therapy. Subsequent articles will address therapeutic translation (Part 2) and selected clinical observations (Part 3). Future research should focus on more approaches and methods including, but not limited to, additional diagnostic biomarkers and tools of artificial intelligence to further improve the possibilities of personalized diabetes care.

## Data Availability

No new data were created or analyzed in this study.
